# Emergence of *Klebsiella pneumoniae* subspecies *pneumoniae* as a cause of septicaemia in pigs in England

**DOI:** 10.1371/journal.pone.0191958

**Published:** 2018-02-22

**Authors:** Cornelia A. Bidewell, Susanna M. Williamson, Jon Rogers, Yue Tang, Richard J. Ellis, Liljana Petrovska, Manal AbuOun

**Affiliations:** 1 Animal and Plant Health Agency (APHA), Rougham Hill, Bury St Edmunds, Suffolk, England; 2 APHA Weybridge, New Haw, Addlestone, Surrey, England; The University of Melbourne, AUSTRALIA

## Abstract

Between 2011 and 2014 outbreaks of septicaemia due to *Klebsiella pneumoniae* subspecies *pneumoniae* (*Kpp*) were diagnosed on thirteen English pig farms. The most consistent features were rapid deaths of pigs from ten-days-old to weaning, seasonal occurrence (May to September), affected farms being outdoor breeding herds and the location of all but one of the outbreaks in the East Anglia region in Eastern England. Molecular characterisation of the outbreak *Kpp* isolates showed that by multilocus sequencing all were sequence type 25 (ST25) of K2 capsular type with a combination of a 4.3kb plasmid (pKPMC25), three phage sequences and the *rmpA* virulence gene. No archived *Kpp* isolates of porcine origin pre-dating 2011 were identified as ST25. In 2013 there was the first detection of an outbreak *Kpp* isolate showing antimicrobial resistance to six antibiotics. Human infection with *Kpp* ST25 has not been reported in the UK.

## Introduction

*Klebsiella pneumoniae* subsp. *pneumoniae* (*Kpp*) is a Gram-negative bacterium within the family Enterobacteriaceae found in the environment and the alimentary tract of animals. Members of the *Klebsiella* genus cause pneumonia and urogenital infections in carnivores and ungulates, mastitis in ruminants and pigs, enterocolitis in rabbits and sporadic septicaemia in a number of species [1,2]. In England, prior to 2011, *Kpp* infection in individual pigs was diagnosed sporadically from submissions of cases of septicaemia, pneumonia or mastitis. *Kpp* was also occasionally isolated from commensal sites and as a contaminant. In 2011, *Kpp* emerged as a cause of outbreaks of septicaemia in pre-weaned pigs and the annual diagnostic rate for septicaemia due to *Kpp* increased in 2011 from zero in the preceding five years to 7.2% of relevant submissions of pre-weaned pigs [3] to Veterinary Investigation Centres (VICs) of the Animal and Plant Health Agency (APHA). This paper describes the clinical and epidemiological details of septicaemia outbreaks in pigs due to *Kpp*, the molecular characterisation of the case and case related *Kpp* isolates and their comparison to both historical and contemporary non-disease associated porcine *Kpp* isolates.

## Materials and methods

The preliminary isolation of *Kpp* from visceral sites of pigs was by culture on sheep blood and MacConkey with salt agars (both Oxoid) aerobically for 24hrs at 37°C. Suspect *Kpp* appeared as mucoid non- haemolytic lactose-fermenting coliforms and API-20E (bioMerieux) was used to confirm the identity of all isolates. The first case *Kpp* isolate was further confirmed by16S ribosomal RNA sequencing. *Kpp* isolates were cryopreserved onto plastic beads retained at -70°C. All isolates were typed by multilocus sequence typing. Genotypic characterisation methods which included whole genome sequencing (WGS) and antimicrobial resistance testing methods employed are described in the technical appendix (S1 File).

Outbreaks of *Kpp* septicaemia were identified by post-mortem examination of at least two pigs found dead from each farm, with isolation of *Kpp* by culture of systemic sites. Laboratory tests allocated to diagnostic carcase submissions to APHA VICs is at the discretion of the investigating veterinary pathologist. Porcine reproductive and respiratory syndrome virus (PRRSV) and influenza virus infections were investigated in pigs from 14 and 8 outbreaks respectively (using methods described in S1 File). Following the first six outbreaks, a case definition was agreed as ‘pigs found dead with lesions consistent with septicaemia and pure or predominant growths of *Kpp* isolated from internal sites in multiple pigs’; these isolates are referred to as ‘case’ isolates (C, n = 25). On three case farms, contemporaneous with the outbreak, healthy cohorts from affected (piglets total n = 20) and unaffected (piglets total n = 26) litters were sampled by swabbing the rectum and oropharynx. The isolates from healthy pigs on case farms and from sows with mastitis on one case farm are referred to as case- related (CR) isolates (n = 17). There were 28 APHA archived *Kpp* isolates from 1990 to 2006 originating from pigs of varying ages and clinical presentations; predominantly pneumonia with only one from a case of septicaemia and one from a pre-weaned pig. These are referred to as historical isolates (n = 28). From July 2011 all isolates of *Kpp* obtained from porcine diagnostic submissions submitted to APHA which were not associated with septicaemia due to *Kpp* were archived. These are referred to as contemporary non-disease associated (CNDA) isolates (n = 62).

### Ethics statement

All sampling of animals described in this paper was done under the UK Veterinary Surgeon’s Act (1966) as part of diagnostic investigation into disease outbreaks on the farms. The dead piglets and sow with mastitis which yielded isolates were not euthanased specifically for this publication and the case-related isolates were from samples collected from live piglets in litters within cohorts of pigs affected with disease as part of the investigation. This sampling strategy is part of the normal veterinary diagnostic investigation of this type of disease outbreak on a farm and as such is not for scientific purpose and therefore not covered by the Animal (Scientific Procedures) Act 1986. Sampling which is for the immediate or long term benefit of the individual animal, its immediate cohort or the wider epidemiological group is covered as an act of veterinary clinical practice within the Veterinary Surgeon’s Act 1966. The sampling (oral cavity and rectal swabs) was undertaken by veterinary surgeons, did not require anaesthesia or euthanasia, and was not harmful to the piglets.

## Results

### Clinical and epidemiological farm data

**Fig 1:**
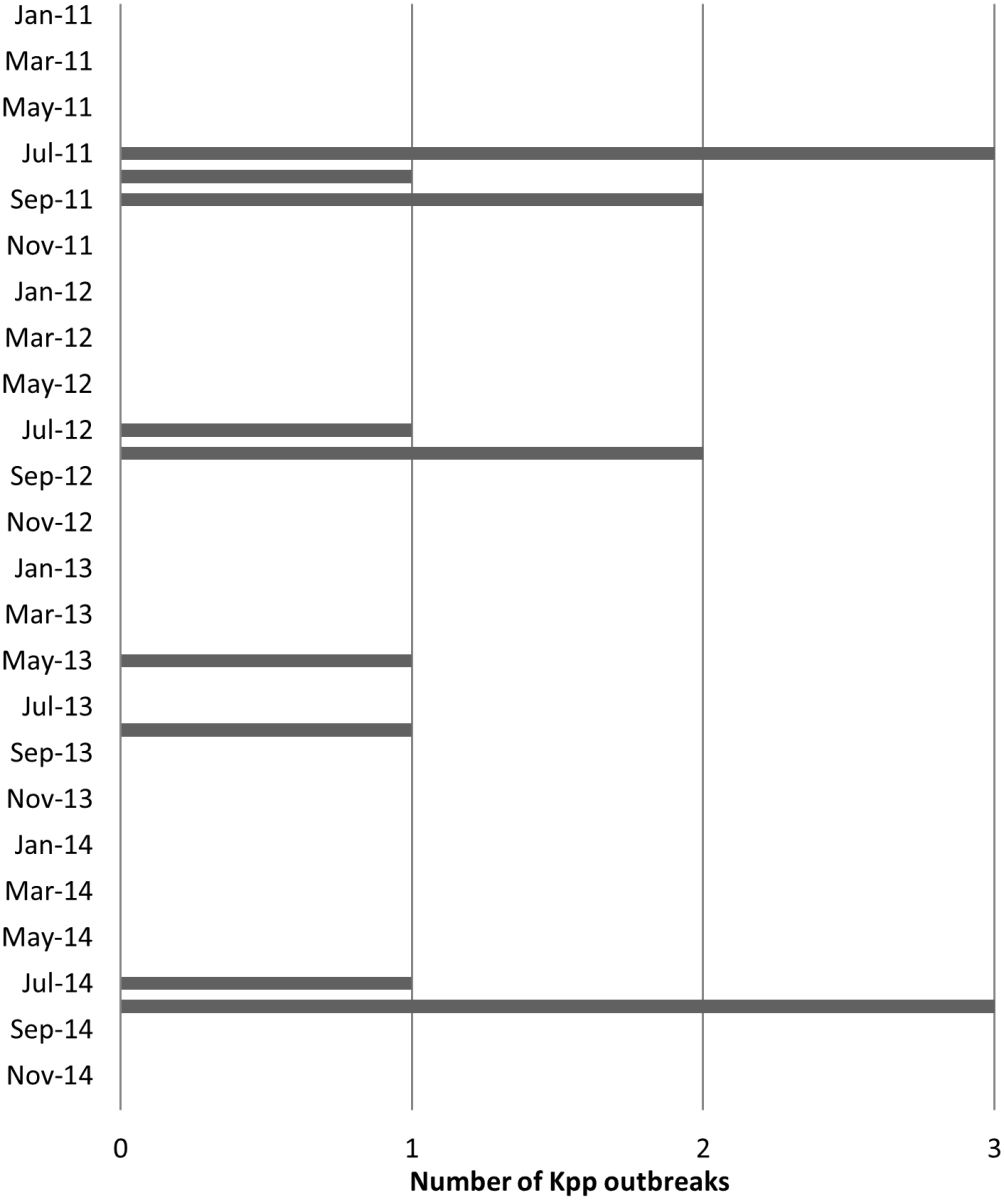
Seasonality of outbreaks of septicaemia due to *Kpp* in pre-weaned pigs. Annual number of outbreaks between May and September.

The first outbreak of septicaemia due to *Kpp* was diagnosed in July 2011 and, by September 2014 fifteen outbreaks were diagnosed on thirteen farms in England (Table 1); two farms had recurrent outbreaks in separate years. The highest annual incidence of outbreaks was six in 2011. All cases occurred in the months May to September (Fig 1). Herd size ranged from 340 to 5000 sows and all pigs affected were commercial hybrid breeds. The rate of litters with perceived cases of *Kpp* septicaemia ranged from 8% to 50% with a mean of 16%. Within litter mortality was usually less than 50% and the loss of one or two piglets per litter was the most common presentation. On one farm the only deaths occurred on a single day in 30- day- old pigs which had been weaned into outdoor pens 36 hours earlier. In all other herds, deaths were in pigs aged 10 to 28 days. Well grown pigs being found dead was the principal presenting clinical sign in all outbreaks. Affected litters were not necessarily in adjacent individual sow farrowing paddocks and where adequate records were kept disease was not recorded in the litters of sows subsequently occupying paddocks which previously held affected litters. Following diagnosis, some private veterinary surgeons implemented control measures as detailed in Table 2.

**Table 1:** Clinical details for English pig herds with outbreaks of septicaemia due to *Kpp* infection.

Case	Type of unit	Piglets indoor or outdoor	Location	Herd parity (P)	Age of piglets (days)	Tooth clipped	Tail docked	Mortality^1^ per batch ^%^	Concurrent disease with PRRSV, SI
1	WP	Outdoor	East Anglia	All 1	12–25	no	no	2 to 5	Nil
2	WP	Outdoor	East Anglia	65% 1–3, 35% 4–6	12–28	no	no	3 to 5	Nil, SI NT
3	WP	Outdoor	East Anglia	ND	28	ND	ND	5.0	NT
4**	WP	Outdoor	East Anglia	1–4 (66% P1)	14–28	no	yes	9.0	Nil, SI NT
5	WP	Outdoor	East Anglia	1–8 (mean 3)	18–25	no	some	2.0	Nil
6	WP	Outdoor	East Anglia	1–6 (mean 3)	14–28	no	yes	3.0	Nil
7	WP	Outdoor	East Anglia	1–5 (mean 2.5)	15–28	no	yes	1.1	Nil
8*	WP	Outdoor	East Anglia	1–4 (mean 2)	21–24	ND	ND	8.3	Nil
9	WP	Outdoor	East Anglia	1–4 (mean 2.5)	18–27	no	no	3.5	Nil
10	BF	Indoor	East Anglia	ND	15–20	yes	yes	2.3	Nil, SI NT
11	WP	Outdoor	East Anglia	1–6 affected sows P4	30	some	yes	2.9	Nil
12	BF	Indoor (breeders indoor and outdoor; affected piglets were born and reared indoors)	South West England	1–7 (mean 3.2)	10–18	yes	no	15.8	Nil, SI NT
13	WP	Outdoor	East Anglia	ND	21	ND	ND	0.8	Nil
14*	WP	Outdoor	East Anglia	ND	21 sows	ND	ND	ND 4.2	Nil NT
15**	WP	Outdoor	East Anglia	1 to 4 (mean 2)	22–23	no	yes	10.3	Nil, SI NT

*and** = repeat cases on the same farm in different years,

^1^mortality attributed by farmer to *Kpp* septicaemia,

WP = weaner producer, BF = breeder finisher, ND = no data, NT = not tested, PRRSV = Porcine reproductive and respiratory syndrome virus, SI = swine influenza

**Table 2:** Clinical interventions and efficacy following the diagnosis of piglet septicaemia due to *Kpp*.

Case	Treatment of pigs during the outbreak	Efficacy	Prophylactic interventions for piglets in subsequent batches	Efficacy	Duration of outbreak
1	Parenteral marbofloxacin 2% solution to cohorts in an affected litter	No further deaths in treated litters	None	NA	June—August 2011
2	a) Parenteral Procaine Benzylpenicillin 30% to cohorts in an affected litter	a) further deaths in treated litters	None	NA	July—September 2011
	b) Parenteral marbofloxacin 2% solution to cohorts in an affected litter	b) No further deaths in treated litters			
3	NK		NK		July 2011—NK
4**	a) Parenteral Procaine Benzylpenicillin 30% to cohorts in an affected litter	NK	None	NA	August 2011—NK
5	None	NA	None	NA	August—October 2011
6	None	NA	None	NA	September 2011 –NK
7	Starter creep feed fed to cohorts in an affected litter	No further deaths in fed litters	Starter creep feed fed to all litters	No further deaths	July—September 2012
8*	Parenteral marbofloxacin 2% solution to cohorts in an affected litter	No further deaths in treated litters	NK	NK	August 2012 one week
9	Parenteral Procaine Benzylpenicillin 30% to cohorts in an affected litter	Further deaths in treated litters	Parenteral Procaine Benzylpenicillin 30% to cohorts in an affected litter	deaths in treated litters	August 2012—NK
10	Parenteral Procaine Benzylpenicillin 30% to cohorts in an affected litter	Further deaths in treated litters	NK	NK	May 2013—NK
11	Weaner feed containing tiamulin and apramycin 36 hours prior to disease outbreak. Amoxicillin Trihydrate in drinking water to cohorts in an affected litter	No further deaths	Medicated weaner feed only	No further deaths	September 2013 one day
12	Parenteral amoxicillin to cohorts in an affected litter	Further deaths in treated litters	Parenteral tulathromycin and iron at four days old	No further deaths	June—July 2013 four weeks
13	NK	NK	NK	NK	Possibly since May 2014 diagnosed August 2014—NK
14*	Sow mastitis treated early in disease with parenteral Trimethoprim and Sulfadiazine	Good recovery	NK	NK	August—September 2014
	Piglets affected were the first batch to receive creep feed, no treatment given	NA	Creep feed medicated from 10 days old with Trimethoprim and Sulfadiazine	No further deaths	
15**	Introduction of creep feed	No further deaths	NK	NK	August—September 2014 one week

NA = not applicable, NK = not known,

*and** = repeat cases on the same farm in different years

### Multilocus sequence typing (MLST) analysis

**Fig 2:**
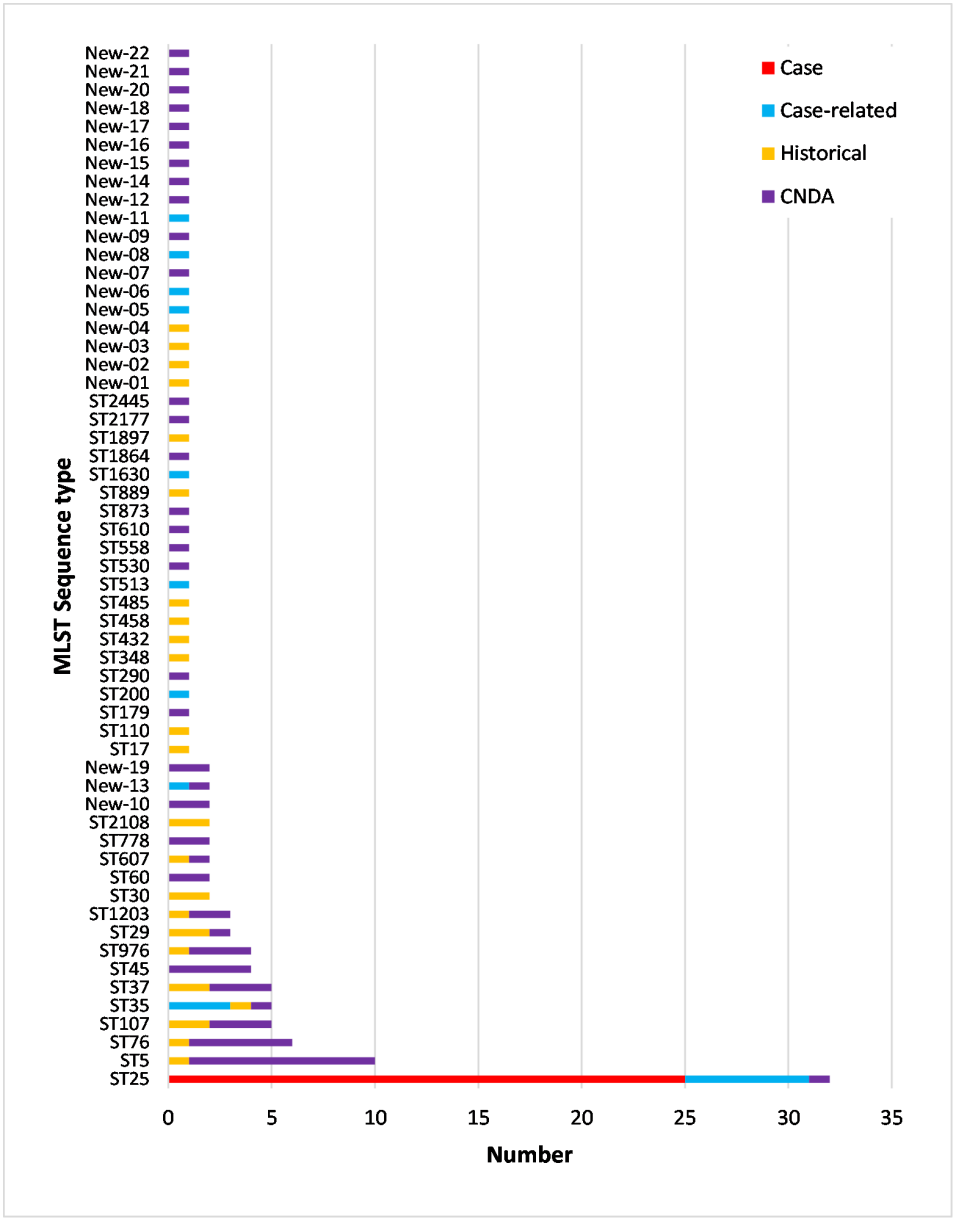
Results of *Kpp* multilocus sequence typing for British historical (1993–2010) isolates, porcine contemporary non-disease associated (CNDA) isolates collected 2011 to 2014 and for outbreak cases (C) and case-related but non cases (CR).

To determine whether the case isolates were genetically related, 132 isolates as detailed above were analysed by MLST [4]. The *Kpp* isolates belonged to 57 different sequence types (ST), 22 of which were ‘new’ types not previously found in the MLST database (Fig 2). All 25 case isolates from septicaemic piglets, with all 15 outbreaks represented, were typed as ST25. A further six of the 17 CR isolates were typed as ST25 these were one from a sow group paddock faeces, three from three separate affected litter healthy cohort piglets’ oral cavity and two from the udders of sows which died of mastitis due to *Kpp*. The 28 historical isolates were sequence typed as 23 different types none of which was ST25, and four of which were 4 ‘new’ STs. The 62 CNDA isolates were sequence typed as 36 different types one of which was ST25 (a 2014 sample) and 17 of which were 14 ‘new’ STs. This single non-case-related CNDA ST25 isolate originated from the colon of a seven-week-old pig with salmonellosis from a rearing farm supplied by a separate breeding farm which had not had *Kpp* septicaemia diagnosed.

### Laboratory findings from case herds

#### Piglets

**Fig 3:**
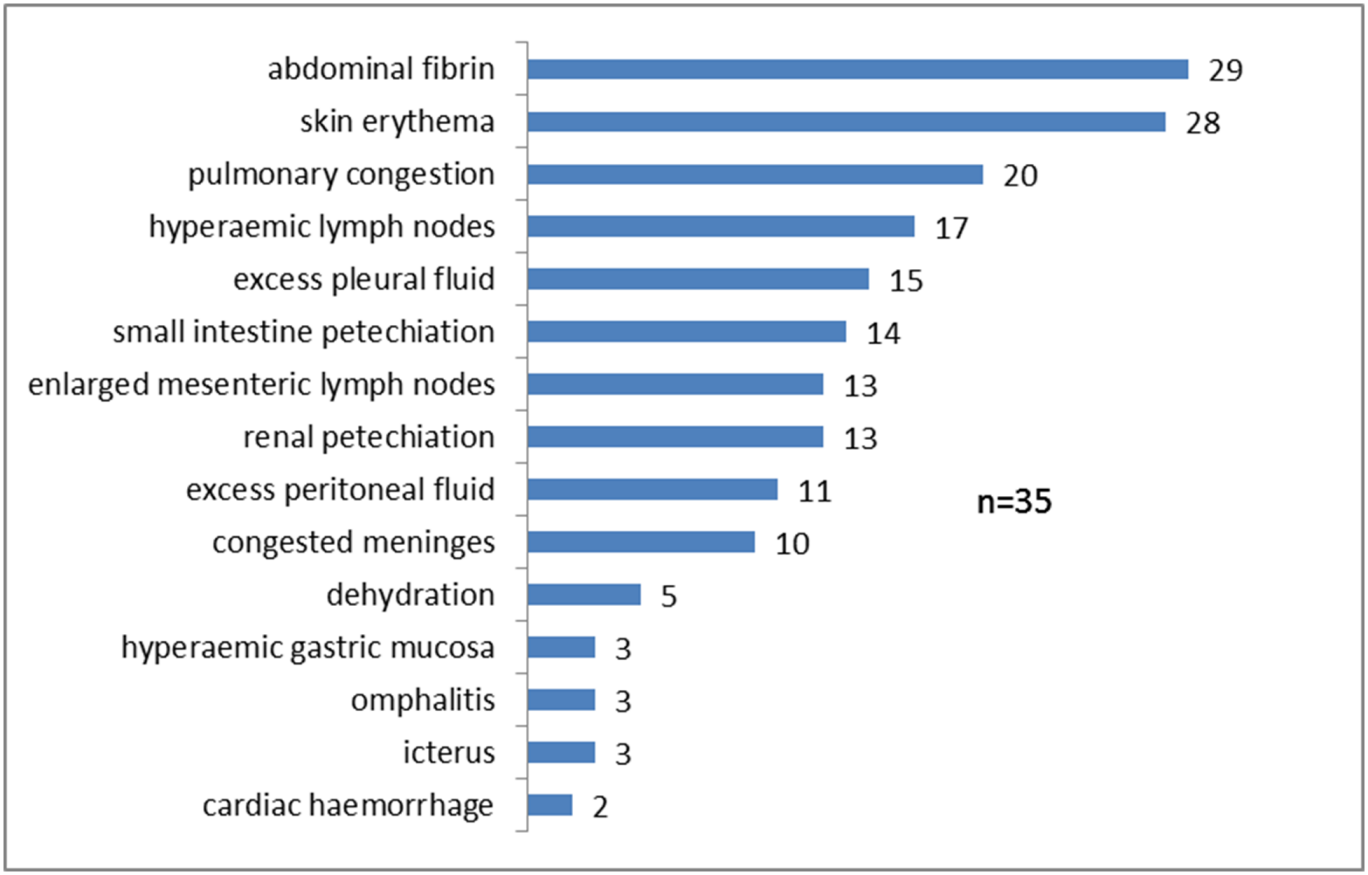
Frequency of gross lesions in thirty five piglets which died of septicaemia due to *Klebsiella pneumoniae*.

All piglets in which death due to *Kpp* septicaemia was diagnosed showed a range of lesions (Fig 3) consistent with those observed with other bacterial septicaemia aetiologies. *Kpp* was readily isolated in pure or predominant growth from multiple visceral sites (brain, liver and lung most commonly). Histological examination of brain from two cases confirmed that despite *Kpp* being cultured from brain there was no associated inflammatory response. No *Kpp* was isolated from faeces of healthy piglets on case farms. On one case farm, oral carriage of *Kpp* ST25 was 44% in healthy piglets in affected litters and zero in healthy piglets from unaffected litters. On the other two case farms, no oral carriage of *Kpp* was detected in healthy cohort pigs.

#### Sows

All farms except one reported that adult pigs were healthy at the time of the outbreak. On one farm in 2014 contemporaneous with the first piglet deaths due to *Kpp* septicaemia, eight sows were affected with severe acute mastitis, with five dying. The cause of mastitis was confirmed as *Kpp* ST25. Affected sows did not necessarily have litters affected with *Kpp* septicaemia.

### Antimicrobial resistance of *Kpp* ST25

Using disc diffusion methodology as described in the technical appendix (S1 File) all *Kpp* ST25 were resistant to ampicillin. One 2013 (BL331, C), one 2014 (BL366, C) and one 2014 (BL364, CNDA) and a sow mastitis isolate (BL357, 2014, CR) were resistant to doxycycline, tetracycline and streptomycin. All these isolates, with the exception of the sow mastitis isolate, were resistant to apramycin and spectinomycin.

### Virulence gene analysis

Virulence genes analyses of 51 *Kpp* isolates using PCR [5] showed that the *rmpA* gene was detected in all the case ST25 isolates analysed (n = 13) (Table 3). Other virulence genes detected in the ST25 isolates included the *mrkD*, *fimH*, *uge*, *wabG*, *ureA*. The *rmpA* gene was also detected in a historical ST5 (BL191) isolate and a new ST (BL201: New-07) isolate from the CNDA collection. The presence of other virulence genes was variable, although all 28 historical, 6 CNDA and 7 case-related were positive for *ureA*. Following testing of the isolates up to 2011, all future *Kpp* submissions were only screened for the presence of *rmpA* gene. All subsequent *Kpp* ST25 isolates associated with the outbreak septicaemia cases between 2012 and 2014 (n = 19) were positive for *rmpA*.

**Table 3:** Virulence gene analysis of 51 isolates from 1993 to 2011.

Isolate type	ID	Year	*magA*	*allS*	*rmpA*	*mrkD*	*kfuBC*	*cf29a*	*fimH*	*uge*	*wabG*	*ureA*	MLST ST
Case	BL142	2011											ST25
Case	BL143	2011											ST25
Case	BL147	2011											ST25
Case	BL193	2011											ST25
Case	BL194	2011											ST25
Case	BL195	2011											ST25
Case	BL202	2011											ST25
Case	BL145	2011											ST25
Case	BL148	2011											ST25
Case	BL151	2011											ST25
Case-related	BL196	2011											ST1630
Case-related	BL197	2011											New-05
Case-related	BL198	2011											New-06
Case-related	BL203	2011											ST25
Case-related	BL204	2011											ST25
Case-related	BL205	2011											New-08
Case-related	BL206	2011											ST25
CNDA	BL199	2011											ST37
CNDA	BL200	2011											ST37
CNDA	BL201	2011											New-07
CNDA	BL207	2011											New-09
CNDA	BL208	2011											New-10
CNDA	BL209	2011											ST179
Historical	BL141	2005											ST107
Historical	BL146	1994											ST485
Historical	BL149	1993											ST17
Historical	BL150	1995											ST889
Historical	BL152	1997											ST29
Historical	BL170	1990											ST110
Historical	BL171	1990											ST37
Historical	BL172	1991											ST37
Historical	BL173	1991											ST432
Historical	BL174	1992											ST1897
Historical	BL175	1992											ST607
Historical	BL176	1992											New-01
Historical	BL177	1993											ST29
Historical	BL178	1993											ST107
Historical	BL179	1993											ST458
Historical	BL180	1998											ST976
Historical	BL181	1994											ST76
Historical	BL182	1994											New-02
Historical	BL183	1994											ST2108
Historical	BL184	1995											ST30
Historical	BL185	1995											ST30
Historical	BL186	1995											ST2108
Historical	BL187	1997											New-03
Historical	BL188	1998											ST348
Historical	BL189	2002											New-04
Historical	BL190	2002											ST35
Historical	BL191	2003											ST5
Historical	BL192	2006											ST1203

Isolates positive for virulence genes by PCR are highlighted in black.

### Plasmid analysis

**Fig 4:**
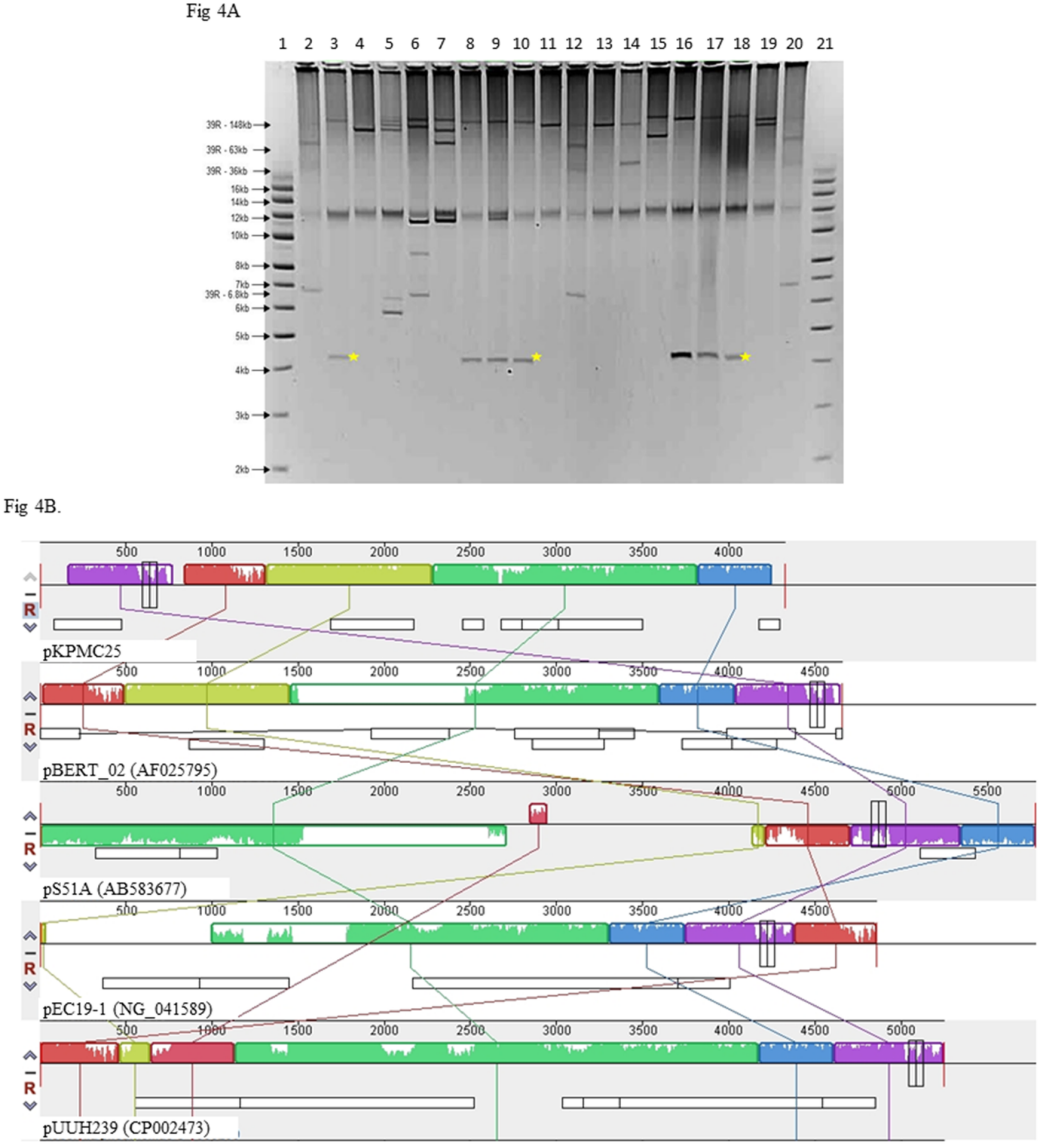
**(A) Gel picture showing electrophoretic separation of plasmids carried by 16 *Kpp* isolates**, migrating in 0.8% agarose (TBE), for 270 min at 150 v, 4°C, are shown. Yellow stars indicate location of pKPMC25 common to *Kpp* ST25 isolates. The plasmids in lane 1 and 21 are supercoiled DNA ladder. Lane 2, 12, 20 shows the reference plasmid bands from strain 39R861 (148 kb, 63 kb, 36 kb, genomic DNA band and 6.8 kb. Lane 3, 8–10 and 16–18 are *Kpp* ST25 isolates. Lanes 4–7, 11, 13–15 and 19 are non-ST25 *Kpp* isolates. (**B). Overview of pKPMC25 plasmid sequence features and comparison to closest matching plasmids**.

Twenty one *Kpp* ST25 and 17 *Kpp* isolates of nine other ST (ST5, 17, 29, 35, 107, 110, 485, 889 and five new ST) were screened for the presence of plasmids. Isolates harboured between one and five plasmids ranging in size from 4kb to 150kb (Fig 4A). A plasmid of 4 kb was unique to all *Kpp* ST25 isolates; no *Kpp* of other ST harboured this plasmid. Sequencing and circularisation of the purified plasmid, using primers KlebVir-25 and KlebVir-26, identified a novel 4325bp plasmid pKPMC25. Annotation of pKPMC25 plasmid identified seven open reading frames (ORFs) (Table 4) encoding three mobilization proteins and four hypothetical proteins. Four plasmids of similar size (4.6 to 5.7kb) deposited in the NCBI database had 75–88% sequence coverage compared to pKPMC25 with 96–98% sequence identity (Fig 4B). Plasmid pBERT_02 (4.6kb) (accession number AF025795) had the closest homology to pKPMC25 with 88% sequence coverage and 96% identity.

**Table 4:** List of 7 features found on pKPMC25.

Feature	Start	Stop	Function	Protein blast
peg1	473	81	mobilization protein MobC	
peg2	2167	1688	hypothetical protein	pBERT_02 [*Salmonella* enterica subsp. *enterica* serovar Berta]/[*Escherichia coli* MS 124–1]
peg3	2573	2454	hypothetical protein	
peg4	2794	2675	hypothetical protein	mobilization protein A/*E*.*coli* O111
peg5	3006	2794	hypothetical protein	mobilization protein D [*Enterobacter cloacae/Klebsiella pneumoniae*]
peg6	3495	3010	MobB	
peg7	4295	4173	mobilization protein MobC	

### Genome sequence analysis

**Fig 5:**
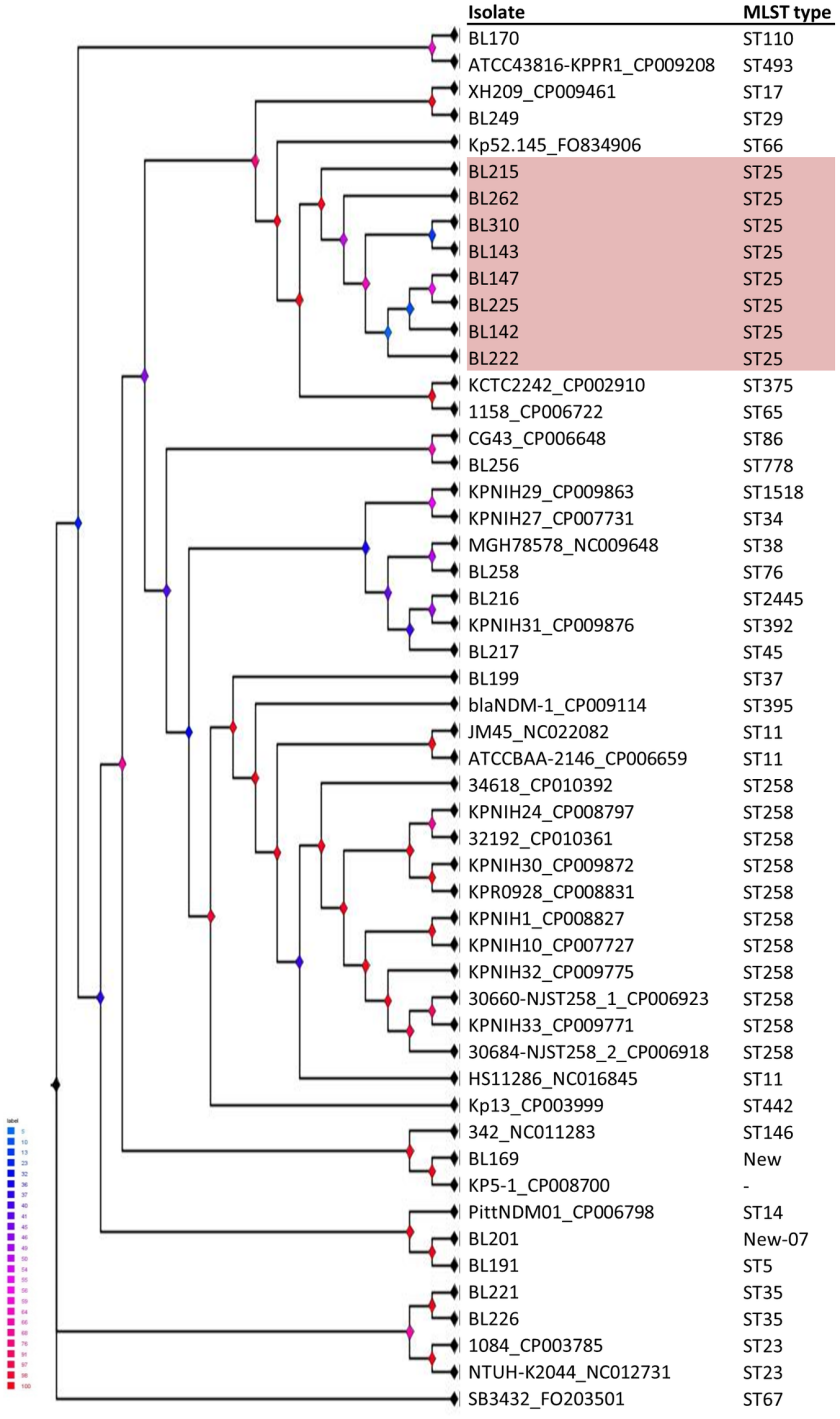
Phylogenetic SNP tree analysis of *Klebsiella* genomes, using *Klebsiella pneumoniae* NTUH-K2044 as reference genome. Shaded isolates the ST25 case isolates. There were a total of 195803 SNP positions in the final dataset. Node colours indicate bootstrap support according to figure legend (Blue = low bootstrap support, Red = high bootstrap support).

**Fig 6:**
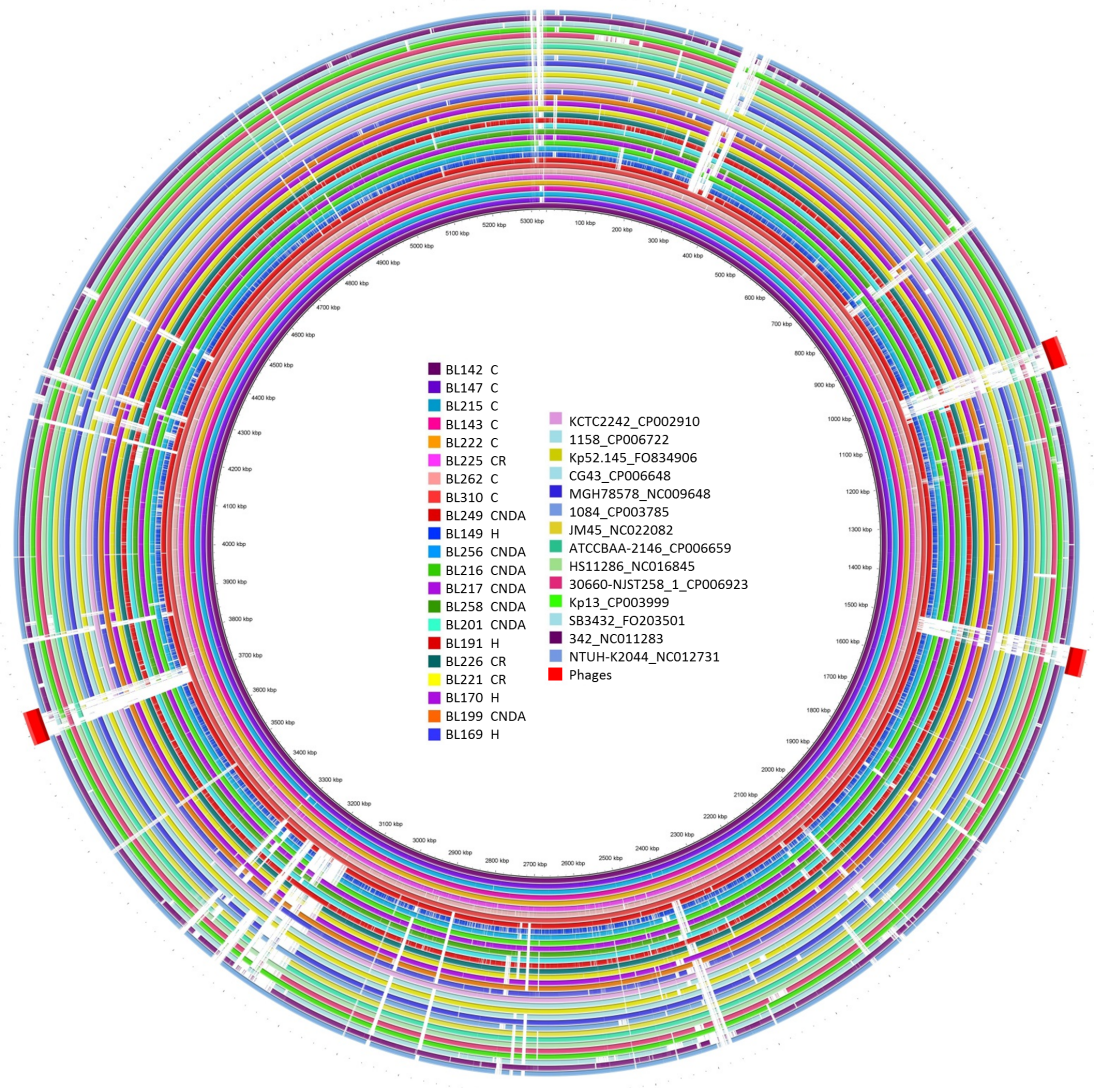
BRIG analysis: Location of genomic regions unique to ST25 case isolates. The 3 phage sequences linked to *Kpp* ST25 isolates are highlighted with red bars. C, case; CR, case-related; cnda, contemporary non disease associated; H, historical.

Whole genome sequences of eight case (C) *Kpp* ST25 isolates (from 2011–2013), three historical *Kpp* (from 1990, 1993 and 2003) and seven non-ST25 *Kpp* CNDA isolates (from 2011–2012) were generated. Thirty three *Kpp* genome sequences deposited in the NCBI database were included in the comparison (accession numbers listed in S1 Table). A phylogenetic tree built using single nucleotide polymorphisms (SNPs) in the core genomes of all analysed strains revealed clear separation of the eight ST25 isolates from the rest of the sequenced *Kpp* strains included (Fig 5). The nearest publically available genome sequence was KCTC2242 (CP002910) of unreported origin, which had 9723 SNP differences compared to the *Kpp* ST25 isolates [6]. The historical and CNDA porcine *Kpp* isolates were distributed between eight SNP groups. BRIG analysis [7] used to compare similarities between a central reference genome (*Kpp* ST25 –isolate BL142) and other *Kpp* genomes identified nine genomic regions unique to *Kpp* ST25 isolates (Fig 6). The nine regions consisted of 121 ORFs, five of which were found to be unique to the ST25 case isolates and were located in two genomic regions (S2 Table). A further 88 ORFs were only associated with the ST25 isolates and were not identified in the non-ST25 porcine isolates, although all of these were found in up to five previously published genomes. In concordance with the SNP analysis, the KCTC2242 genome shared the highest number of these unique ORFs with ST25 isolates (46 of 88 ORFs). Fifty four percent of the 121 ORF identified in the *Kpp* ST25 isolated were annotated as bacteriophage genes such as phage proteins, tail fibre proteins and bacteriophage integrase genes indicating potential phage acquisition by the ST25 case isolates. The phage finding tool PHAST [8] was used to identify potentially intact bacteriophages within the sequenced genomes. Three intact phage sequences were identified in the ST25 case isolates ranging in size between 43.4kb-53.3kb (Fig 6). A phage located at 1006827-1054152bp, consisting of 71 ORFs, had the highest homology to 17 genes found in pYD38_A isolated from Vibrio isolate (NC_021534) (Fig 6) as well as 16 genes from Salmon_E1 phage (NC_010495) and Cronob_ENT47670 (NC_019927) phages. The two other intact phage sequences, Fels_2 (NC_010463) located at 1500902-1544354bp and TL_2011b (NC_019445) located at 3683896-3737248bp found in the case *Kpp* ST25, were also found with varying low homology (21.6% to 79.4%, mean 34.6% average) in the non-ST25 *Kpp* historical and CNDA isolates sequenced.

The capsule is a major virulence factor of *Kpp* and has been used as the basis of a typing scheme [9–11] separating *Kpp* into 77 capsular (K) types. The K types of these sequenced *Kpp* were determined by BLAST analysis. Ten capsular types were detected, with all the ST25 case isolates belonging to K2, which is associated with human invasive disease [12] (Table 5). Among the non-ST25 isolates, BL170 (ST110) was identified as K2, BL217 (ST45) was identified as K5 and BL249 (ST29) had a K54, all of which are capsular types linked to invasive disease. The K-types (K14, K22, K28, K39, K51, K58, and K61) found in the other non- ST25 isolates have not previously been associated with pathogenicity.

**Table 5:** Comparison of virulence determinants in ST25 *Klebsiella pneumoniae* isolates to other *K*. *pneumoniae* from porcine cases.

ID	BL142	BL143	BL147	BL215	BL215	BL222	BL262	BL310	BL221	BL225	BL226	BL199	BL201	BL216	BL217	BL249	BL256	BL258	BL149	BL170	BL191
**EBI (ENA) sequence Accession number**	ERS1069962	ERS1069963	ERS1069964	ERS1069965	ERS1069965	ERS1069966	ERS1069968	ERS1069969	ERS1080571	ERS1069967	ERS1080572	ERS1080567	ERS1080568	ERS1080569	ERS1080570	ERS1080573	ERS1080574	ERS1080575	ERS1080564	ERS1080565	ERS1080566
**Isolate type**	Case								Case Related			Contemporary Non-Disease Associated							Historical		
**Host**	Porcine																				
**Year**	2011	2011	2011	2012	2012	2012	2012	2013	2012	2012	2012	2011	2011	2012	2012	2012	2012	2012	1993	1990	2003
***rmpA***	+	+	+	+	+	+	+	+	-	+	-	-	+	-	-	-	-	-	-	-	+
**MLST ST**	ST25	ST25	ST25	ST25	ST25	ST25	ST25	ST25	ST35	ST25	ST35	ST37	New-07	ST2445	ST45	ST29	ST778	ST76	ST17	ST110	ST5
**4.5kb plasmid**	+	+	+	+	+	+	+	+	-	+	-	-	-	-	-	-	-	-	-	-	-
**Capsular Type**	K2	K2	K2	K2	K2	K2	K2	K2	K22	K2	K22	K14	K39	K28	K5	K54	K51	K61	K58	K2	K39
**fyuA_6,Receptor for yersiniabactin transport**																					
**iroB_6,Salmochelin**																					
**iroC_11,Salmochelin**																					
**iroD_6,Salmochelin**																					
**iroN_5,Salmochelin**																					
**irp1_1,yersiniabactin**																					
**irp2_13,yersiniabactin**																					
**rmpA_11,Mucoid phenotype**																					
**ybtA_5,Yersiniabactin system**																					
**ybtE_7,Yersiniabactin system**																					
**ybtP_6,Yersiniabactin system**																					
**ybtQ_9,Yersiniabactin system**																					
**ybtS_8,Yersiniabactin system**																					
**ybtT_7,Yersiniabactin system**																					
**ybtU_6,Yersiniabactin system**																					
**ybtX_7,Yersiniabactin system**																					
**KPHS_14360,enterochelin synthetase component D**																					
**KPHS_14380,enterochelin esterase**																					
**KPHS_02860,outer membrane ferric enterobactin receptor**																					
**KPHS_14370,ferrienterobactin receptor**																					
**KPHS_14410,iron-enterobactin transporter ATP-binding protein**																					
**KPHS_14430,ferric enterobactin transport protein**																					
**KPHS_14440,enterobactin exporter EntS**																					
**KPHS_14450,iron-enterobactin transporter periplasmic binding protein**																					
**KPHS_14470,enterobactin synthase subunit E**																					
**KPHS_30860,ferric enterobactin transporter ATP-binding protein**																					
**KPHS_41620,outer membrane porin for ferric Enterobactin & colicins B and D**																					
**BL142_Phage_pYD38_A NC021534 ph1 1006827–1054152**																					
**BL142_Phage_Fels_2 NC010463 ph2 1500902–1544354**																					
**BL142_Phage_TL_2011b NC019445 ph3 3683896–3737248**																					

Values greater than 80% are considered as gene presence. The shaded cells represent the percentage presence of gene where black = 100%, dark grey = 75%, medium grey = 50%, light grey = 25% and white = 0%.

An extended virulence gene search of the 20 *Kpp* genome sequences using 80 genes previously identified in virulent clones of *Kpp*, identified several additional virulence factors associated with the ST25 outbreak *Kpp* isolates. The iron enterobactin siderophore system (*entBEF*) was common to all sequenced isolates including non-ST25 isolates. In addition to the regulator of mucoid phenotype gene (*rmpA*), all the ST25 isolates harboured three siderophore systems: Salmochelin (*iroBCDN*), two yersiniabactin systems (*ybtAEPQSTUX-fyuA* and *irp1*,*irp2*) and iron enterobactin system (*entBEF*) (14) (Table 5). Two non-ST25 isolates (BL191, BL201) showed a similar siderophore profile to the ST25 isolates. Seven of the non-ST25 isolates (BL149, BL170, BL216, BL226, BL249, BL249 and BL256) lacked both the Salmochelin (*iroBCDN*) and the two yersiniabactin systems (*ybtAEPQSTUX-fyuA* and *irp1*, *irp2*) that were identified in the ST25 isolates. In addition, three CNDA isolates (BL199, BL217 and BL258) lacked the salmochelin siderophore system as well as the *rmpA* genes.

## Discussion

The emergence of *Kpp* ST25 as a cause of outbreaks of septicaemia in pigs in England is supported by the consistent detection of this strain in all outbreaks since 2011, and its absence from archived porcine *Kpp* isolates dating back to 1993 and from all but one of the CNDA *Kpp* isolated since 2011. Historically *Kpp* has been a cause of sporadic infections in pigs, including septicaemia, pneumonia and mastitis [13] but the occurrence of multiple outbreaks with group mortality up to 16% is a new manifestation of disease due to *Kpp*. There are several consistent features about the outbreaks; disease is strictly seasonal and occurs in predominantly outdoor pre-weaned piglets from 10 days old. These features suggest that unidentified factors relating to pig susceptibility, environment or management are involved in the pathogenesis of disease. About forty percent of an estimated 6,000 breeding herds in England are outdoors, with many of these being in East England [14,15]. All 13 affected herds have over 100 sows; there are 810 farms with 100 or more sows, representing nearly 90% of the national breeding herd population.

No epidemiological link was found between the single outbreak in South West England and those in East England. Although pig disease surveillance is undertaken across England, this is a passive surveillance system and a number of factors may influence submissions and the derived disease surveillance data. It is possible that low-level mortality due to *Kpp* would not be investigated by APHA and the number of outbreaks diagnosed may underestimate how widespread disease due to *Kpp* ST25 is. From 2011 awareness of the occurrence and clinical characteristics of *Kpp* outbreaks was raised amongst pig farmers and veterinary practitioners [16]. It is unlikely that many outbreaks with the level of mortality occurring in those described in this paper would not be investigated, particularly as disease occurs in the later preweaning period as unexpected deaths of pigs in good body condition. In cattle, mastitis due to *Kpp* has a causal association with wood shavings or sawdust [17]. The bedding on pig units in England is almost exclusively straw and outbreak farms were no different. Farmers were specifically asked if more mastitis was being seen than usual. Only one unit reported any mastitis concurrent with piglet septicaemia and this was diagnosed as being due to *Kpp* ST25, thus there is no evidence that clinical disease in lactating sows was the source of infection for piglets in most outbreaks. Live pigs, in particular, replacement breeding pigs, were considered as a possible means of introducing the *Kpp* ST25 strain to herds, however there was no common source herd or source pyramid for affected farms and two of the affected herds had received no pigs from external sources for two and five years. There were no obvious epidemiological links common to affected farms to explain the emergence of disease, although the concentration of cases in East Anglia raises the possibility of regional spread through fomites, birds or other means. The maximum and minimum distances between East Anglian case farms was 100 km and less than 2 km respectively. There was no concurrent disease identified in pigs which died of septicaemia due to *Kpp*. Concurrent infection with PRRSV, which commonly precipitates other diseases in pigs, was ruled out in piglets on the 14 case farms where it was investigated. On one case farm healthy piglets from litters which had deaths due to *Kpp* were carrying *Kpp* ST25 in the oropharynx but, although the number of piglets sampled was small, oropharyngeal carriage of *Kpp* ST25 was not demonstrated on the two other farms sampled. One sow faeces on a case farm yielded *Kpp* ST25 indicating, not unexpectedly, that pig faeces is a potential source of infection. Faecal excretion of *Kpp* ST25 was also demonstrated in one CNDA sample from a weaned pig with no links to an outbreak farm. Interventions were implemented to control disease in 11 outbreaks and included antimicrobial treatment of remaining piglets in affected litters, the introduction of un-medicated or medicated creep feed prior to weaning and treatment of neonatal piglets with a combination of antimicrobial and iron. Some interventions during the outbreak have shown cessation or reductions in mortality (parenteral marboflaxacin on three farms, and introduction of creep feed on two farms). As no control groups of pigs were monitored without the intervention, efficacy cannot be assessed and the disease was self-limiting on farms without any intervention. Interestingly although multiple antibiotic resistance is a feature of human *Kpp* isolates [18], most of the case isolates were susceptible to a panel of 13 antimicrobials. All case isolates had the expected intrinsic resistance to ampicillin and indeed following parenteral penicillin or amoxicillin use on four farms piglet deaths continued reflecting this intrinsic resistance. In 2013 and 2014, 3 isolates of *Kpp* ST25 from three separate farms showed resistance to an additional five antimicrobials by disc diffusion testing and surveillance is continuing to monitor whether antimicrobial resistance (AMR) emerges in *Kpp* isolates derived from APHA submissions and to investigate the genetic basis for the acquired AMR.

In Australia *Kpp* has emerged as a cause of septicaemia in pigs aged one to four weeks with three premises affected between 2014 to 2016. The Australian outbreaks were in the summer months and two were due to ST25 with a concurrent ST1978 and ST278 infection in the third herd. In contrast to the cases in England which largely occurred in outdoor pigs these Australian cases were all in indoor pigs. [19].

Several molecular analyses were used to determine the genetic relatedness of the case *Kpp* strains as well as their genetic relationship to other *Kpp* isolates found in porcine samples. MLST analyses indicated that all the case *Kpp* isolates were sequence type ST25. At the time of this study there were only six ST25 isolates, all from human sources, submitted to the MLST database which contains 2956 *Kpp* isolates from across the globe and which represent 73 different sequence types [20]. Of the six human ST25 isolates, one was isolated from urine (Brazil) and the other five were found in blood (Poland, France and Vietnam). Initial molecular analyses identified unique genome features associated with all porcine ST25 outbreak isolates; the presence of the *rmpA* virulence gene and a 4.3kb plasmid (pKPMC25). A PCR for rapid identification of *Kpp* isolates harbouring pKPMC25 was designed by APHA using primers based on the sequence of the ORF2 that had homology to an ORF (hypothetical protein) in a pBERT_2 from *Salmonella* Berta (accession number: AF025795.1). The pBERT_2 is a Colicigenic plasmid that was identified in *S*. enterica Berta which appeared to have a role in its pathogenesis. The presence of the *rmpA* gene, a regulator of mucoid phenotype was common, although not unique, to outbreak *Kpp* strains. This virulence gene has been linked with human invasive disease [5,18]; there was little or no difference in the mucoid phenotype of the outbreak isolates. The presence of additional genotypic traits was investigated using WGS analysis and identified several additional factors which could contribute to the virulent phenotype observed in the *Kpp* ST25 disease outbreaks. The thick polysaccharide capsular types, such as K1, K2, K5, K54 and K57, of *Klebsiella* have been associated with pathogenicity or invasive disease. The sequenced *Kpp* ST25 isolates had K2 capsular types and could be distinguished from non-ST25 isolates by the presence of three siderophore systems (Salmochelin, yersiniabactin and enterobactin) and three phage sequences. In one study [5], *Kpp* ST25 isolates (including ST65, ST243 and ST66) were found to belong to clonal complex CC65^K2^ and were associated with severe infections in animals (cat, monkey) and human clinical infections (isolated from abscess and blood). The study also demonstrated the virulent nature (in murine infection model) of CC65^K2^ clonal group although this was dependent on the presence of one or more virulence genes. However, their *Kpp* ST25 isolate (Poland isolated in 1997) differed to the ST25 from this study with the absence of the *rmpA* gene. A recent study which included four *Kpp* ST25 isolated from human invasive infections, but had K72 capsule types (a different capsular type to the porcine *Kpp* ST25 isolates), also reported the presence of the yersiniabactin and salmonchelin siderophore systems as well as the mucoid phenotype regulator *rmpA* virulence genes [18]. Another determinant which could play a role in the pathogenesis of the porcine *Kpp* ST25 isolates is the presence of three intact phage sequences. Bacteriophages have been associated with severe infection such as the shiga toxin producing *E*.*coli* (STEC) which harbours a phage encoding the main virulence determinant of STEC isolates [21]. There is no known human disease in England associated with these outbreaks of septicaemia due to *Kpp* in pigs. These analyses indicate the emergence of a virulent ST25 *Klebsiella pneumoniae* in pre-weaned pigs in England.

## Conclusion

Clinical and epidemiological evidence supported by genetic analyses, shows the emergence of a particular *Kpp* strain, ST25, which appears to be novel and virulent in pigs and has caused seasonal outbreaks of septicaemia in pre-weaned pigs annually in England since 2011.

## Supporting information

S1 File Technical appendix. (PDF)

S1 Table *Kpp* genome sequences deposited in the NCBI database isolates included in the SNP analysis. (PDF)

S2 Table Open reading frames unique to ST25 outbreak isolates. (PDF)
