# Supplementary material for: Emergence of Klebsiella pneumoniae subspecies pneumoniae as a cause of septicaemia in pigs in England
Source: PLoS One. 2018 Feb 22;13(2):e0191958. doi: 10.1371/journal.pone.0191958 (PMC5823397; doi:10.1371/journal.pone.0191958)
Supplement: S1 File — (PDF) [file pone.0191958.s001.pdf]

S1 File. Technical appendix

Real-time reverse transcription polymerase chain reaction (RRT-PCR) for detection of influenza A virus.

RNA was extracted from a pool of tonsil, lung and trachea by an automated programme in a Universal BioRobot (Qiagen, UK) [1]. RRT-PCR testing of the RNA extracts was performed using the "perfect match" matrix gene assay for generic detection of swine influenza virus [2]. All amplifications were carried out in an Mx3005P Sequence Detection System (Agilent).

PRRSV PCR

RNA was isolated from serum using the Roche MagNA Pure robot (Roche Diagnostics Ltd, Burgess Hill, UK), and PRRSV RNA detected by quantitative real-time reverse transcription PCR (qRT-PCR) using the QIAGEN QuantiTect® Probe RT-PCR kit (Qiagen, Hilden, UK) [3].

Antimicrobial susceptibility testing

Twenty two *Kpp* ST25 isolates (representing 1 isolate from each of 22 submissions) were tested by standard disc diffusion method for trimethoprim / sulphamethoxazole (25µg), ampicillin (10µg), florfenicol (30µg), enrofloxacin (5µg), doxycycline (30µg), tetracycline (10µg), apramycin (15µg), spectinomycin (25µg), neomycin (10µg), streptomycin (10µg), amoxicillin / clavulanic acid (30µg), cefpodoxime (10µg) and ceftiofur (30µg). Interpretation was by zone size breakpoints applied to veterinary bacteria, derived from the British Society for Antimicrobial Chemotherapy susceptibility testing guidelines or determined by APHA [4].

Bacterial PCR analysis

Crude bacterial DNA lysates were prepared for each isolate, using a 5µl loopful of bacteria cultured on 5% sheeps blood agar (SBA) into 500µl of sterile DNase and RNase free water. This was vortexed to create a homogenous suspension and subsequently boiled for 15 minutes at 96°C. The tube was centrifuged at 13000 rpm for 5 minutes to remove cellular debris and the DNA containing supernatant transferred to a fresh tube. Two microliters of the lysate was used as template in subsequent PCR reactions. All PCRs were conducted on a GeneAmp PCR System 9700 (Applied Biosystems) in a total volume of 20 ml with final concentrations of 1.5 mM MgCl2 and 200 mM each dNTP, 1.0 U Taq DNA polymerase and 2µl of bacterial DNA lysate (HotStarTaq Plus Master Mix kit, Qiagen). The thermal cycling conditions were as follows: an initial denaturation at 95 ^{o}C for 10 min, followed by 30 cycles at 95 ^{o}C for 30 s, Tm for 45 s and 72 ^{o}C for 1 min, completed by a final extension for 10 min at 72 ^{o}C. The presence of PCR amplicons was examined by electrophoresis on a 1.5% agarose gel along with a 100 bp and a 1 kb DNA Ladder (Promega) as markers. The following primer sets were used to characterise isolates.

Virulence gene analysis: The presence of 10 virulence genes was assessed as described by Brisse et al [5].

Multilocus sequence typing: Multilocus sequence typing was carried out as previously described by Diancourt et al [6] and as detailed on the *Kpp* MLST Database website [7]. The seven housekeeping MLST loci (rpoB, gapA, mdh, pgi, phoE, infB, and tonB) from all isolates were analysed by PCR and ABI sequencing. Data analysis was set up using Seqscape (Applied Biosystems) software to align

forward and reverse sequencing reads to a reference and allows contigs to be built. Consensus sequences were then compared to the *Kpp* MLST Database to assign allelic profiles and sequence type (ST).

pKPMC25 plasmid analysis: The presence of the pKPMC25 plasmid was determined by PCR using primers KlebP-Peg2-F1 (GTCGGAAACCAGATTTCGAC) and KlebP-Peg2-R1 (GTGAACTTGTTACTTCCACG) directed towards the hypothetical protein homologous to a gene in *Salmonella berta* plasmid (pBERT_2). The reactions were performed with an annealing temperature of 58°C and an extension time of 1 minute. The resulting product was 600bp.

Plasmid analysis

Plasmid profiling*:* Plasmid DNA was extracted from the isolates using the alkaline lysis method of Kado and Liu [8]. DNA was separated on 0.8% agarose gel in 89 mM Tris-borate and 2 mM EDTA, pH 8.3 (TBE) buffer and visualised under a UV transluminator. The plasmids were sized by inclusion of a reference *E. coli* strain 39R861 which carries plasmids of 147kb, 63kb, 37kb and 7kb [9] and a supercoiled DNA ladder (D5292, Sigma-Aldrich).

Plasmid Sequencing*:* Plasmid DNA was purified using the Qiagen miniprep extraction kit according to manufacturer's instructions. To determine purity, plasmid DNA was visualised on 1% TBE agarose gel. A DNA library was prepared from 500 ng of plasmid DNA following Roche guidelines and sequenced using a Roche 454 GS-FLX system. Sequences were assembled using Newbler version 2.3 (Roche),

which resulted in 2 contigs of 4098bp and 180 bp and a depth of 100 was achieved. Single contigs were closed by PCR and ABI sequencing.

Genome sequencing and analysis

DNA was extracted from 3 ml overnight cultures using Gentra Puregene Yeast/Bac kit B as per manufacturer's instructions (Qiagen, UK). Sequencing was performed by the APHA Central Sequencing Unit, Weybridge. The Illumina GAIIx platform was used to produce paired-end libraries for 20 *Kpp* isolates which included 8 ST25 isolates from outbreak cases, following manufacturer's instructions. Sequences were assembled de novo using Newbler v2.5. FASTQ data is available at European Nucleotide Archive under Study accession number: PRJEB12817. The short reads were quality trimmed with Trimmomatic and mapped to the reference (the reference we used to compare) using BWA [10]. The Sequence Alignment Map output from BWA was sorted and indexed to produce a Binary Alignment Map (BAM) using Samtools [11]. Freebayes [12] was used to create a Variant Call Format (VCF) file from each of the BAMs, which were further parsed to extract only single nucleotide polymorphism (SNP) with the minimum number of reads covering the variant position being 10 and the minimum proportion of those reads which must differ from the reference being 0.9. Pseudosequences of polymorphic positions were used to create maximum likelihood phylogenetic trees using RAxML[13,14]. The trees were visualised using FigTree v1.4.2 [15]. RAST was used annotate the sequenced Kpp genomes [16]. The BLAST Ring Image Generator (BRIG) was used to generate a comparison of the overview of Klebsiella genomes in order to identify DNA sequence linked to the outbreak ST25 isolates [17]. The PHAST (PHAge Search Tool) webserver was used for the identification of phage elements in the *Kpp* genomes [18].

The presence of virulence genes, genes unique to ST25 and capsular K types [19] was determined using BLAST [20].

References

1. Slomka MJ, Pavlidis T, Coward VJ, Voermans J, Koch G, *et al*. (2009) Validated Real Time reverse transcriptase PCR methods for the diagnosis and pathotyping of Eurasian H7 avian influenza viruses. Influenza Other Respir Viruses 3: 151-164.
2. Slomka MJ, Densham AL, Coward VJ, Essen S, Brookes SM, *et al*. (2010) Real time reverse transcription (RRT)-polymerase chain reaction (PCR) methods for detection of pandemic (H1N1) 2009 influenza virus and European swine influenza A virus infections in pigs. Influenza Other Respir Viruses 4: 277-293.
3. Frossard JP, Fearnley C, Naidu B, Errington J, Westcott DG, *et al*. (2012) Porcine reproductive and respiratory syndrome virus: antigenic and molecular diversity of British isolates and implications for diagnosis. Vet Microbiol 158: 308-315.
4. UK Veterinary Antibiotic Resistance and Sales Surveillance, UK-VARSS 2013, https://www.gov.uk/government/uploads/system/uploads/attachment_data/file/440744/VARSS.pdf
5. Brisse S, Fevre C, Passet V, Issenhuth-Jeanjean S, Tournebize R, *et al*. (2009) Virulent clones of *Klebsiella pneumoniae*: identification and evolutionary scenario based on genomic and phenotypic characterization. PLoS One 4: e4982.
6. Diancourt L, Passet V, Verhoef J, Grimont PA, Brisse S (2005) Multilocus sequence typing of *Klebsiella pneumoniae* nosocomial isolates. J Clin Microbiol 43: 4178-4182.
7. MLST Database website, www.pasteur.fr/recherche/genopole/PF8/mlst/Kpneumoniae.html
8. Kado CI, Liu ST (1981) Rapid procedure for detection and isolation of large and small plasmids. J Bacteriol 145: 1365-1373.
9. Threlfall EJ, Rowe B, Ferguson JL, Ward LR (1986) Characterization of plasmids conferring resistance to gentamicin and apramycin in strains of *Salmonella typhimurium* phage type 204c isolated in Britain. J Hyg (Lond) 97: 419-426.
10. Li H, Durbin R (2010) Fast and accurate long-read alignment with Burrows-Wheeler transform. Bioinformatics 26: 589-595.
11. Li H, Handsaker B, Wysoker A, Fennell T, Ruan J, *et al*. (2009) The Sequence Alignment/Map format and SAMtools. Bioinformatics 25: 2078-2079.
12. Garrison E, Marth G (2012) Haplotype-based variant detection from short-read sequencing. arXiv:12073907 [q-bioGN]

13. Stamatakis A (2014) RAxML version 8: a tool for phylogenetic analysis and post-analysis of large phylogenies. Bioinformatics 30: 1312-1313.
14. Silvestro D, Michalak I (2012) raxmlGUI: a graphical front-end for RAxML. Org Divers Evol 12.
15. Figtree v1.4.2, http://tree.bio.ed.ac.uk/software/figtree/
16. Aziz RK, Bartels D, Best AA, DeJongh M, Disz T, *et al*. (2008) The RAST Server: rapid annotations using subsystems technology. BMC Genomics 9: 75.
17. Alikhan NF, Petty NK, Ben Zakour NL, Beatson SA (2011) BLAST Ring Image Generator (BRIG): simple prokaryote genome comparisons. BMC Genomics 12: 402.
18. Zhou Y, Liang Y, Lynch KH, Dennis JJ, Wishart DS (2011) PHAST: a fast phage search tool. Nucleic Acids Res 39: W347-352.
19. Pan YJ, Lin TL, Chen YH, Hsu CR, Hsieh PF, *et al*. (2013) Capsular types of *Klebsiella pneumoniae* revisited by wzc sequencing. PLoS One 8: e80670.
20. Tao T (2010) Standalone BLAST Setup for Windows PC. BLAST® Help [Internet]: Bethesda (MD): National Center for Biotechnology Information (US).
