# Supplementary material for: Emergence of Klebsiella pneumoniae subspecies pneumoniae as a cause of septicaemia in pigs in England
Source: PLoS One. 2018 Feb 22;13(2):e0191958. doi: 10.1371/journal.pone.0191958 (PMC5823397; doi:10.1371/journal.pone.0191958)
Supplement: S1 Table — (PDF) [file pone.0191958.s002.pdf]

S1 Table. ***Kpp*** **genome sequences deposited in the NCBI database isolates included in the SNP analysis.**

Isolate	Accession number	MLST ST	Publication title
ATCCBAA-2146	CP006659	11	Resistance Determinants and Mobile Genetic Elements of an NDM-1-Encoding Klebsiella pneumoniae Strain
HS11286	NC016845	11	Complete genome sequence of Klebsiella pneumoniae subsp. pneumoniae HS11286, a multidrug-resistant strain isolated from human sputum
JM45	NC022082	11	Comparative genomics analysis of aminoglycoside resistance in multi-drug resistant Klebsiella pneumoniae JM45 isolate
1084	CP003785	23	Complete Genome Sequence of Klebsiella pneumoniae 1084, a Hypermucoviscosity-Negative K1 Clinical Strain
MGH78578	NC009648	38	Reduced susceptibility to carbapenems in Klebsiella pneumoniae clinical isolates associated with plasmid-mediated beta-lactamase production and OmpK36 porin deficiency.
Kp52.145	FO834906	66	Genomic Definition of Hypervirulent and Multidrug-Resistant Klebsiella pneumoniae Clonal Groups
SB3432	FO203501	67	PCR-based identification of Klebsiella pneumoniae subsp. rhinoscleromatis, the agent of rhinoscleroma
CG43	CP006648	86	Complete genome sequence of Klebsiella pneumoniae CG43, a K2 serotype liver abscess isolate
342	NC011283	146	Complete genome sequence of the N2-fixing broad host range endophyte Klebsiella pneumoniae 342 and virulence predictions verified in mice
30660-NJST258_1	CP006923	258	Molecular dissection of the evolution of carbapenem-resistant multilocus sequence type 258 Klebsiella pneumoniae
30684-NJST258_2	CP006918	258	Molecular dissection of the evolution of carbapenem-resistant multilocus sequence type 258 Klebsiella pneumoniae
KCTC2242	CP002910	375	Complete Genome Sequence of the 2,3-Butanediol-Producing Klebsiella pneumoniae Strain KCTC 2242
Kp13	CP003999	442	Comparative analysis of the complete genome of KPC-2-producing Klebsiella pneumoniae Kp13 reveals remarkable genome plasticity and a wide repertoire of virulence and resistance mechanisms
KPNIH31	CP009876	392	Single-molecule sequencing to track plasmid diversity of hospital-associated carbapenemase-producing Enterobacteriaceae
KP5-1	CP008700	-	Complete Genome Sequence of a Klebsiella pneumoniae Strain Isolated from a Known Cotton Insect Boll Vector (southern green stink bug)
1158	CP006722	65	Complete Genome Sequence of Klebsiella pneumoniae 1158, a K2 clinical strain
32192	CP010361	258	Human Excreted bodily substance, 2010
34618	CP010392	258	Human Bodily fluid 2011
ATCC43816-KPPR1	CP009208	493	Complete Genome Sequence of Klebsiella pneumoniae Strain ATCC 43816 KPPR1, a Rifampin-Resistant Mutant Commonly Used in Animal, Genetic, and Molecular Biology Studies
blaNDM-1	CP009114	395	Surveillance of carbapenem-resistant Klebsiella pneumoniae: tracking molecular epidemiology and outcomes through a

			regional network
KPNIH1	CP008827	258	Tracking a hospital outbreak of carbapenem-resistant Klebsiella pneumoniae with whole-genome sequencing
KPNIH10	CP007727	258	Tracking a hospital outbreak of carbapenem-resistant Klebsiella pneumoniae with whole-genome sequencing
KPNIH24	CP008797	258	Tracking a hospital outbreak of carbapenem-resistant Klebsiella pneumoniae with whole-genome sequencing
KPNIH27	CP007731	34	Whole genome sequencing of Klebsiella pneumoniae
KPNIH29	CP009863	1518	Single-molecule sequencing to track plasmid diversity of hospital-associated carbapenemase-producing Enterobacteriaceae
KPNIH30	CP009872	258	Single-molecule sequencing to track plasmid diversity of hospital-associated carbapenemase-producing Enterobacteriaceae
KPNIH32	CP009775	258	Single-molecule sequencing to track plasmid diversity of hospital-associated carbapenemase-producing Enterobacteriaceae
KPNIH33	CP009771	258	Single-molecule sequencing to track plasmid diversity of hospital-associated carbapenemase-producing Enterobacteriaceae
KPR0928	CP008831	258	Whole genome sequencing of Klebsiella pneumoniae
PittNDM01	CP006798	14	Whole-Genome Assembly of Klebsiella pneumoniae Coproducing NDM-1 and OXA-232 Carbapenemases Using Single-Molecule, Real-Time Sequencing
XH209	CP009461	17	Complete Genome Sequence of Klebsiella pneumoniae Sequence Type 17, a Multidrug-Resistant Strain Isolated during Tigecycline Treatment
NTUH-K2044	NC012731	23	Genome sequencing and comparative analysis of Klebsiella pneumoniae NTUH-K2044, a strain causing liver abscess and meningitis
