# Supplementary material for: Emergence of Klebsiella pneumoniae subspecies pneumoniae as a cause of septicaemia in pigs in England
Source: PLoS One. 2018 Feb 22;13(2):e0191958. doi: 10.1371/journal.pone.0191958 (PMC5823397; doi:10.1371/journal.pone.0191958)
Supplement: S2 Table — (PDF) [file pone.0191958.s003.pdf]

S2 Table. Open reading frames unique to ST25 outbreak isolates.

Open reading frame	Location in BL142 genome
BL142_SC_peg.2359_L3708309-3708103_hypothetical_protein	BRIG_3640-3760
BL142_SC_peg.2369_L3715134-3714994_hypothetical_protein	BRIG_3640-3760
BL142_SC_peg.2373_L3717665-3717531_hypothetical_protein	BRIG_3640-3760
BL142_SC_peg.4566_L1022426-1023640_hypothetical_protein	BRIG_960-1080
BL142_SC_peg.4567_L1024536-1024733_hypothetical_protein	BRIG_960-1080
